# Outcomes of restorative proctectomy and ileal pouch-anal anastomosis: a matched-cohort analysis of robot-assisted, laparoscopic, and open surgery

**DOI:** 10.1007/s00464-026-13300-8

**Published:** 2026-08-24

**Authors:** Tommaso Violante, Stefano Cardelli, Angela Romano, Argentina Tiano, Giacomo Calini, Gilberto Poggioli, Marco Novelli, Matteo Rottoli

**Affiliations:** 1 Surgery of the Alimentary Tract, IRCCS Azienda Ospedaliero-Universitaria Di Bologna, Bologna, Italy; 2 Department of Medical and Surgical Sciences, Alma Mater Studiorum—University of Bologna, Via Massarenti 9, 40138 Bologna, Italy; 3 Division of Colon and Rectal Surgery, Mayo Clinic, Rochester, MN USA; 4 Department of Statistical Sciences, Alma Mater Studiorum—University of Bologna, Bologna, Italy

**Keywords:** Robotic surgery, IPAA, Ulcerative colitis, Robotic

## Abstract

**Background:**

Ileal pouch-anal anastomosis (IPAA) remains the gold standard for restorative proctocolectomy, yet its technical complexity has limited widespread minimally invasive adoption. Robotic surgery was introduced to overcome the ergonomic and visualization challenges of laparoscopy, but comparative evidence among open, laparoscopic, and robot-assisted IPAA in tertiary care remains scarce.

**Methods:**

A matched-cohort analysis was performed on adult patients undergoing proctectomy with IPAA for ulcerative and indeterminate colitis (January 2005–September 2025) at a tertiary referral center. Patients were stratified by surgical approach: open, laparoscopic, and robot-assisted. The robot-assisted cohort included only cases performed with the modular Hugo™ RAS platform. Primary endpoints were total operative time and 30-day postoperative surgical complications. Multivariable regression, propensity score matching, and G-computation were applied to adjust for confounders such as age, BMI, ASA class, disease duration, and biologic exposure.

**Results:**

Among 764 patients, 573 underwent open, 145 laparoscopic, and 46 robot-assisted IPAA. Adjusted analyses demonstrated no significant differences in 30-day surgical complications between robot-assisted and laparoscopic (OR 1.56, 95% CI 0.63–3.90) or open (OR 1.40, 95% CI 0.57–3.44) approaches. Both minimally invasive techniques yielded faster bowel function recovery (− 1.2 days vs open, *p *< 0.001) and reduced hospital stay (− 4.1 days for robot-assisted, − 4.7 days for laparoscopic; *p *< 0.05). Operative time was longer in minimally invasive groups but similar between robot-assisted and laparoscopic cases.

**Conclusion:**

In expert centers, open, laparoscopic, and robot-assisted completion IPAA demonstrate preliminary safety, feasibility, and short-term equivalence. Minimally invasive approaches enhance postoperative recovery, and modern robotic platforms achieve procedural efficiency comparable to laparoscopy, although larger multicenter cohorts are needed to confirm equivalence for rare postoperative outcomes.

Restorative proctocolectomy with ileal pouch-anal anastomosis (IPAA), first introduced by Parks and Nicholls in 1978, is the gold standard surgical treatment for many patients with ulcerative and indeterminate colitis. It offers a cure from the underlying disease while preserving intestinal continuity [1, 2]. The procedure, however, remains one of the most technically demanding in colorectal surgery, requiring precise dissection deep within the narrow confines of the pelvis and is associated with a considerable complication rate [3, 4].

To mitigate these challenges and improve patient outcomes, surgical techniques have evolved from traditional open laparotomy toward minimally invasive approaches. Laparoscopy marked the first significant leap forward, establishing a new standard of care by reducing postoperative pain, shortening hospital stays, and improving cosmetic results [5–8]. Despite these advancements, conventional laparoscopy has inherent limitations, such as rigid instrumentation and a two-dimensional view, which can amplify the difficulty of pelvic surgery.

Starting from the early 2000s robotic platforms were specifically developed to overcome these hurdles by providing surgeons with wristed instruments, 3D high-definition vision, and enhanced ergonomics. Given these advantages, the adoption of robotic surgery in the colorectal field has grown considerably [9, 10] and has been rapidly applied to IPAA. Despite this trend, there is a lack of comprehensive analyses from major tertiary institutions comparing the outcomes of open, laparoscopic, and robotic approaches [11, 12].

This study aims to fill that gap. Using data from our specialized, high-volume tertiary IBD referral center, we provide a comprehensive analysis of the surgical results for open, laparoscopic, and robot-assisted IPAA, including our initial experience with a novel modular robotic platform. By comparing key perioperative outcomes, we seek to better define the role of this new technology in modern IPAA surgery.

## Methods

### Surgical cohorts and data collection

This retrospective study included patients who underwent proctectomy with IPAA from January 2005 to September 2025 at our tertiary referral center. The study protocol was reviewed and approved by the Institutional Review Board (IRB) to ensure compliance with ethical standards and protection of patient data. Laparoscopic IPAA was introduced in our center in 2016, while the Hugo RAS system was adopted in 2024. At our institution, we do not utilize ERAS/ERP protocols for IBD patients, as current literature data have not shown these protocols to yield a reduction in postoperative complications for this specific population [13]. However, all patients were managed under a standardized institutional post-proctectomy with IPAA clinical care protocol. The nasogastric (NG) tube placed at induction was routinely removed at procedure end or on Postoperative Day (POD) 1 upon awakening, unless severe nausea or distension warranted extended decompression. At the end of the procedure, a transanal Foley catheter was routinely inserted through the pouch-anal anastomosis for luminal decompression and removed on POD 1–2. Urinary catheters were removed on POD 1–2, provided hemodynamic stability was present and no nerve/urological repairs occurred. Pelvic drains were monitored daily and removed on POD 3–4 when output fell below 50 mL/24 h without signs of deep infection. Oral clear fluids were initiated on POD 1–2 upon the presence of stoma output (flatus/stool) and advanced as tolerated. Pain management relied on multimodal non-opioid analgesia paired with patient-controlled analgesia (PCA) or epidural analgesia, transitioning to oral agents. Low-molecular-weight heparin (LMWH) thromboprophylaxis was initiated on POD 1 and continued for up to 30 days postoperatively.

Patients were stratified into three cohorts based on the surgical approach: open IPAA, multiport laparoscopic IPAA, or robot-assisted IPAA. The robot-assisted cohort exclusively included procedures performed using the Hugo™ RAS modular robotic system (Medtronic, Minneapolis, MN, USA). We excluded patients affected by Familial Adenomatous Polyposis or Crohn’s Disease, as well as those undergoing a 1-stage or traditional 2-stage IPAA. Our study exclusively includes patients undergoing a completion proctectomy with IPAA. While the surgical approach (open, laparoscopic, or robotic) differed during the completion proctectomy phase, ileal pouch creation (extracorporeal J-pouch) and double-stapled ileal pouch-anal anastomoses were standardized across all three cohorts. Restricting the analysis to completion proctectomies specifically isolates the comparative effect of the surgical platform on the deep pelvic rectal dissection phase, eliminating procedural noise and variability associated with abdominal colectomy and pouch configuration. Including upfront total proctocolectomies would introduce significant variability in total operative time, blood loss, and morbidity related to the abdominal colectomy phase.

Data were extracted from a prospectively maintained institutional inflammatory bowel disease (IBD) surgery database. Demographic and preoperative variables collected comprised age, sex, Body Mass Index (BMI), smoking status, the American Society of Anesthesiologists (ASA) classification, primary diagnosis (ulcerative colitis [UC], indeterminate colitis [IC]), surgical approach in first-stage colectomy, and preoperative corticosteroid or biologic agent use within three months prior to IPAA surgery.

Intraoperative and technical variables recorded included total operative time from skin incision to closure, intraoperative complications, use of mesenteric lengthening techniques, pouch creation method, anastomosis type (stapled or hand-sewn), number of surgical drains placed, and type of diverting loop ileostomy constructed.

Postoperative outcomes within 30 days were thoroughly documented, including length of hospital stay (LOS) and times to first fluid intake, solid diet, and bowel movement. A diverting loop ileostomy (DLI) was created in all patients across the study. For these diverted patients, the metric “time to first bowel movement” specifically represents the time to first stoma output (flatus or stool).

Complications were graded per Clavien–Dindo classification and categorized as medical (e.g., cardiac events, venous thromboembolism, pneumonia, acute renal failure) or surgical (e.g., anemia requiring transfusion, surgical site infection, intraluminal bleeding, IPAA anastomotic leak, abscess, peritonitis, ileus, or partial small bowel obstruction [pSBO]). Ileus or pSBO was defined as intolerance to liquids for more than 3 days or nausea and vomiting requiring nasogastric tube placement. Additionally, 30-day hospital readmission, reoperation, and conversion to open surgery (in laparoscopic and robot-assisted cohorts) were recorded.

### Study outcomes

The primary endpoints were total operative time and 30-day surgical complication rate. Secondary endpoints included overall 30-day surgical and medical complication rates, conversion to open surgery, and length of hospital stay. Remaining variables were considered tertiary outcomes.

### Surgical techniques

All surgeries were performed by six senior colorectal surgeons experienced in open, advanced laparoscopic, and robot-assisted procedures.

In the open approach, a midline laparotomy was used for completion proctectomy and rectal mobilization, followed by ileal pouch creation. The pouch-anal anastomosis was fashioned using a double-stapled, unless mucosectomy and hand-sewn anastomosis were necessary.

The laparoscopic approach, standardized per prior description [14], involved intracorporeal completion proctectomy. The rectal stump was transected, and the specimen extracted through a Pfannenstiel incision using an Alexis port. The ileal pouch was created extracorporeally, and a double-stapled pouch-anal anastomosis was performed.

The robot-assisted technique utilized the Hugo™ RAS system with a multi-quadrant, two-docking strategy as described previously [15]. The robotic platform was employed for the entire intra-abdominal portion including proctectomy, and small bowel dissection followed by undocking for extracorporeal pouch creation and double-stapled anastomosis, mirroring laparoscopic steps.

All pouches were J pouches, with a median length of 15 cm. Rectal dissection was performed routinely with a total mesorectal excision approach.

In all cases, small bowel mobilization was performed to the uncinate process of the pancreas with preservation of the ileocolic vessels and arcade. Mesenteric lengthening was routinely employed for hand-sewn anastomoses but selectively for stapled IPAA in cases of short mesentery (prior surgery, obesity, abnormal anatomy) or anal stump dehiscence.

Mesenteric lengthening was predominantly achieved by dividing the superior mesenteric vessels distal to the origin of the ileocolic vessels, provided these had been preserved during the previous colectomy. If additional lengthening was required (e.g., to achieve a hand-sewn anastomosis), our preferred supplementary technique was peritoneal scoring [16]. Adequate pouch reach for a tension-free stapled anastomosis was confirmed using the symphysis pubis border as a landmark, which is particularly relevant in obese patients with a bulky mesentery.

The technique involved identifying and clamping the superior mesenteric artery/vein immediately distal to the ileocolic vessel origin, confirming distal ileal perfusion via the ileocolic arcade. All pouches were J-configured (median length 15 cm, body folded anteriorly with the mesentery in the back for ~ 1 cm extra length). The pouch apex required to reach the superior pubic symphysis for tension-free anastomosis.

In the three groups, the same technique was used to transect the rectum (linear 30 mm open TA stapler at 1, 1.5 cm above the dentate line), as previously described [14], which allowed us to consistently obtain a residual rectal cuff shorter than 2.5 cm for double-stapled anastomosis. A diverting loop ileostomy (DLI) was created in all cases at the end of the procedure.

### Statistical analysis

Statistical analysis was performed using R software (Version 4.5.1). A *p* value of < 0.05 was considered statistically significant.

Descriptive statistics were used to summarize patient demographics and clinical characteristics; continuous variables were reported as median and interquartile range (IQR), while categorical variables were presented as counts (*n*) and percentages (%).

To compare outcomes between the three surgical cohorts (robot-assisted vs. open, robot-assisted vs. laparoscopic, and laparoscopic vs. open), four distinct analytical approaches were employed to ensure robustness and control for confounding. First, unadjusted (Raw) comparisons were made. Second, multivariable models (Multi) were constructed, using logistic regression for binary outcomes (e.g., complications, reoperation) and linear regression for continuous outcomes (e.g., duration of surgery, length of stay), utilizing robust standard errors. Third, Propensity Score Matching (PSM), using nearest neighbor matching, was performed to create balanced cohorts. Fourth, G-computation (g-comp) was used as an additional causal inference method, with confidence interval derived from bootstrap-based techniques [17]. All adjusted analyses (Multi, PSM, g-comp) controlled for the following covariates: Body Mass Index (BMI), age, gender, presence of comorbidities, history of previous abdominal surgery, steroid use at the time of surgery, preoperative biologic use, duration of disease (years), and ASA score. Outcomes are reported as Odds Ratios (OR) for binary variables and beta coefficient (*β*) for continuous variables, both with 95% confidence intervals (95% CI).

## Results

The study included 764 patients undergoing ileal pouch-anal anastomosis (IPAA): 46 robot-assisted, 145 laparoscopic, and 573 open procedures. Baseline characteristics, as reported in Table 1, showed a median age of 37, 43, and 42 years respectively. Patients in the robotic group had slightly lower BMI (median 22.3 vs 23.0 lap and 23.5 open) and a higher proportion of preoperative biologic use (55.6%) than both laparoscopic (33.8%) and open groups (8.5%).

**Table 1:** Patients’ characteristics

	Robotic (*N* = 46)	Laparoscopic (*N *= 145)	Open (*N *= 573)
Diagnosis:			
- Ulcerative colitis	46 (100)	143 (98.6)	537 (93.7)
- Indeterminate colitis		2 (1.4)	36 (6.3)
Sex ratio (M:F)	24:22	79:66	342:231
Age (years)	37 (25)	43 (14)	42(23)
BMI (kg/m^2^)	22.3 (5)	23 (5)	23.5 (6)
Surgical approach at the subtotal colectomy:			
Open	0 (0)	5 (3.5)	525 (91.6)
Laparoscopic	45 (97.8)	139 (96.5)	48 (8.4)
Robotic	1 (2.2)	0 (0)	0 (0)
Years of disease	8 (11)	7 (10)	7 (2)
ASA score:			
- 1	1 (4.3)	5 (3.4)	33 (5.8)
- 2	39 (84.8)	127 (87.6)	462 (80.6)
- 3	5 (10.9)	18 (12.4)	75 (13.1)
- 4	0	0	3 (0.5)
Steroids within 4 weeks from surgery	2 (4.4)	8 (5.7)	28 (4.9)
Previous or current use of biologics	25 (55.6)	48 (33.8)	45 (7.9)
Operative time (minutes)	250 (75)	230 (75)	215 (75)
Conversion	1 (2.2)	18 (12.4)	
Type of IPAA anastomosis:			
- Stapled	46 (100)	141 (97.9)	548 (95.5)
- Hand-sewn	0 (0)	4 (2.1)	25 (4.5)
Intraoperative surgical complication	0 (0)	3 (2.1)	14 (2.5)
30-day postoperative medical complication	5 (11.1)	15 (10.5)	68 (11.9)
30-day postoperative surgical complication	11 (23.9)	20 (14.2)	97 (16.9)
30-day reoperation	2 (4.3)	6 (4.1)	26 (4.6)
Postoperative day of first fluid intake	1 (1)	2 (1)	4 (0.7)
Postoperative day of first food intake	2 (1)	3 (1)	5 (0.9)
Postoperative day of first bowel movement	2 (1)	3 (1)	4 (0.7)
DLI closure	23 (50)	123 (84.8)	473 (82.5)
DLI closure technique:			
- Hand-sewn	6 (13)	95 (65.5)	397 (69.4)
- Stapled	17 (36.9)	28 (19.3)	81 (14.1)
30-day complication after DLI closure	2 (8.7)	12 (9.8)	39 (6.8)

Categorical variables are number and %; continuous variables are expressed as median and interquartile range, *ASA* American Society of Anesthesiologists, *DLI* diverting loop ileostomy

Median operative time was 250 min (IQR 75) for robot-assisted, 230 (IQR 75) for laparoscopic, and 215 (IQR 75) for open procedures. Conversion to open surgery occurred in 1 (2.2%) robot-assisted and 18 (12.4%) laparoscopic cases. Intraoperative complications were rare across all groups (0% robot-assisted, 2.1% laparoscopic, and 2.5% open). Recorded intraoperative events across laparoscopic and open cohorts included presacral or pelvic sidewall venous plexus bleeding managed with hemostasis, minor seromucosal enterotomies during small bowel mobilization due to dense adhesions from prior subtotal colectomy, and focal staple line incompleteness requiring oversewing. Zero intraoperative surgical complications occurred in the robotic group; the single conversion to open surgery was prompted by extensive freeze-pelvis adhesions rendering robotic dissection unsafe. A stapled anastomosis was performed in 100% of robot-assisted, 97.9% of laparoscopic, and 95.5% of open cases. Mesentery lengthening was performed in 19 open cases and in 5 laparoscopic cases, with no robot-assisted procedure requiring lengthening procedures. Peritoneal scoring was used in a total of 11 cases, ligation of the ileocolic vessels in 9 cases and ligation of the superior mesenteric vessels in the remaining 4 cases. Unadjusted 30-day postoperative outcomes showed comparable rates of medical complications (11.1% vs 10.5% vs 11.9%) and reoperation (4.3% vs 4.1% vs 4.6%) (Table 2). The unadjusted 30-day surgical complication rate was 23.9% for robot-assisted, 14.2% for laparoscopic, and 16.9% for open. Minimally invasive approaches showed faster recovery, with a median time to first food intake of 2 days for robot-assisted and 3 for laparoscopic, compared to 5 for open.

**Table 2:** Detailed breakdown of 30-day postoperative complications

Complication type	Clavien–Dindo grade	Robot-assisted (*n* = 46)	Laparoscopic (*n* = 145)	Open (*n *= 573)
Total surgical complications	Overall	11 (23.9%)	20 (14.2%)	97 (16.9%)
Ileus/partial small bowel obstruction	I–II	8 (17.3%)	12 (8.3%)	60 (10.4%)
Surgical site infection	I–II	1 (2.2%)	3 (2.1%)	15 (2.6%)
Hemorrhage/bleeding	II–III	1 (2.2%)	3 (2.1%)	14 (2.4%)
Anastomotic leak	III–IV	1 (2.2%)	2 (1.4%)	8 (1.4%)
Total medical complications	Overall	5 (11.1%)	15 (10.5%)	68 (11.9%)
Cardiac event	II–IV	1 (2.2%)	3 (2.1%)	14 (2.4%)
Venous thromboembolism	II–III	1 (2.2%)	2 (1.4%)	12 (2.1%)
Pneumonia	II–III	2 (4.4%)	6 (4.1%)	25 (4.4%)
Acute renal failure	II–IV	1 (2.2%)	4 (2.8%)	17 (3.0%)

After adjusting for baseline covariates (including Body Mass Index, age, gender, presence of comorbidities, history of previous abdominal surgery, steroid use at the time of surgery, preoperative biologic use, duration of disease, and ASA score), analysis of primary outcomes showed no significant differences in 30-day surgical complications between the three cohorts (robot assisted vs. Open OR: 1.40, 95% CI [0.57, 3.44], *p *= 0.466; robot assisted vs. Laparoscopic OR: 1.56, 95% CI [0.63, 3.90], *p *= 0.339) (Tables 2, 3, and 4). Similarly, rates of 30-day medical complications and reoperation were not significantly different between any of the groups (robot-assisted vs. open medical OR: 0.85, 95% CI [0.27, 2.70], *p *= 0.786; robot-assisted vs. open reoperation OR: 0.49, 95% CI [0.11, 2.18], *p *= 0.353).

**Table 3:** Comparison of outcomes for robotic-assisted vs. open proctectomy with ileal pouch-anal anastomosis

Robotic vs. Open				
Duration of surgery (minutes)	Beta	*p* val	LL	UL
Raw	**43.5608**	**< 0.001**	**25.1515**	**61.9702**
Multi	**43.3820**	**< 0.001**	**22.7842**	**63.9797**
g-comp	**43.3820**	**< 0.001**	**23.3203**	**65.6497**
PSM	**42.4839**	**< 0.001**	**19.3953**	**65.5724**

Postoperative medical complication	OR	*p* val	LL	UL
Raw	0.9216	0.8682	0.3098	2.2176
Multi	0.8463	0.7830	0.2242	2.5345
g-comp	0.8522	0.7860	0.2686	2.7040
PSM	0.9128	0.8821	0.2401	2.8325

Postoperative surgical complication	OR	*p* val	LL	UL
Raw	1.5450	0.2315	0.7260	3.0587
Multi	1.4124	0.4769	0.5111	3.5119
g-comp	1.3985	0.4656	0.5680	3.4430
PSM	1.9375	0.2107	0.6607	5.3451

Postoperative day of first bowel movement	Beta	*p* val	LL	UL
Raw	− **1.1415**	**< 0.001**	**− 1.5807**	**− 0.7023**
Multi	**− 1.1759**	**< 0.001**	**− 1.7326**	**− 0.6193**
g-comp	**− 1.1759**	**< 0.001**	**− 1.6489**	**− 0.6409**
PSM	**− 1.2647**	**< 0.001**	**− 1.8440**	**− 0.6855**

Length of Hospital Stay (days)	Beta	*p* val	LL	UL
Raw	− 1.9589	0.7337	− 13.2626	9.3447
Multi	− 4.1323	0.5745	− 18.5842	10.3196
g-comp	**− 4.1323**	**0.0430**	**− 10.0093**	**− 0.6234**
PSM	− 5.4762	0.3855	− 17.9133	6.9609

Reoperation	OR	*p* val	LL	UL
Raw	0.9441	0.9388	0.1491	3.3042
Multi	0.4899	0.5178	0.0252	2.9284
g-comp	0.4946	0.3528	0.1120	2.1833
PSM	0.7426	0.7951	0.0362	5.3063

Complication after DLI closure	OR	*p* val	LL	UL
Raw	1.6786	0.4967	0.2614	6.0742
Multi	1.4630	0.7330	0.0744	9.1834
g-comp	1.4427	0.7116	0.2067	10.0670
PSM	1.5312	0.7397	0.0644	17.7119

Bold indicates statistically significant results (*p *< 0.05)

*Raw* Unadjusted comparison, *Multi* Multivariable models, *PSM* Propensity score matching, G-computation, *OR* odds ratio, *p val* *p* value, *LL* lower level, *UL* upper level

**Table 4:** Comparison of outcomes for robotic-assisted vs. laparoscopic proctectomy with ileal pouch-anal anastomosis

Robotic vs. Laparoscopic				
Duration of surgery (minutes)	Beta	*p* val	LL	UL
Raw	4.0014	0.7210	− 18.0994	26.1022
Multi	10.9105	0.4018	− 14.7633	36.5843
g-comp	10.9105	0.1926	− 12.8545	36.0537
PSM	11.3548	0.4059	− 15.6542	38.3639

Postoperative medical complication	OR	*p* val	LL	UL
Raw	1.0667	0.9061	0.3305	2.9487
Multi	1.1041	0.8792	0.2740	3.7288
g-comp	1.0964	0.8594	0.3960	3.0351
PSM	1.3778	0.6431	0.3263	5.2615

Postoperative surgical complication	OR	*p* val	LL	UL
Raw	1.9014	0.1275	0.8113	4.2937
Multi	1.5904	0.3828	0.5350	4.4124
g-comp	1.5633	0.3388	0.6258	3.9048
PSM	1.9375	0.2493	0.6165	5.9888

Postoperative day of first bowel movement	Beta	*p* val	LL	UL
Raw	− 0.0645	0.7630	− 0.4860	0.3570
Multi	0.0031	0.9903	− 0.4964	0.5026
g-comp	0.0031	0.4956	− 0.5177	0.5745
PSM	− 0.0588	0.8413	− 0.6403	0.5226

Length of Hospital Stay (days)	Beta	*p* val	LL	UL
Raw	2.7810	0.1741	− 1.2408	6.8028
Multi	− 0.3842	0.6650	− 2.1335	1.3652
g-comp	− 0.3842	0.2806	− 1.7855	0.8170
PSM	− 0.5286	0.4123	− 1.8020	0.7448

Reoperation	OR	*p* val	LL	UL
Raw	1.0530	0.9506	0.1506	4.7609
Multi	0.4757	0.5410	0.0216	3.9244
g-comp	0.4918	0.2017	0.1654	1.4620
PSM	0.6569	0.7236	0.0306	5.4580

Complication after DLI closure	OR	*p* val	LL	UL
Raw	1.8730	0.4310	0.2797	7.4865
Multi	0.7072	0.7791	0.0308	5.9301
g-comp	0.7320	0.7239	0.1297	4.1329
PSM	0.4688	0.5069	0.0224	3.1220

*Raw* Unadjusted comparison, *Multi* Multivariable models, *PSM* Propensity score matching, G-computation, *OR* odds ratio, *p val* *p* value, *LL* lower level, *UL* upper level

Regarding operative duration, adjusted models indicated that both robot-assisted (*β *+ 43.38 min, 95% CI [23.32, 65.65], *p *< 0.001) and laparoscopic (*β *+ 41.26 min, 95% CI [26.06, 55.33], *p *< 0.001) approaches had significantly longer operative times compared to the open approach. No significant difference was found between robot-assisted and laparoscopic durations (*β *+ 10.91 min, 95% CI [− 12.85, 36.05], *p *= 0.193) (Table 5).

**Table 5:** Comparison of outcomes for laparoscopic vs. open proctectomy with ileal pouch-anal anastomosis

Laparoscopic vs. Open				
Duration of surgery (minutes)	Beta	*p* val	LL	UL
Raw	**39.5594**	**< 0.001**	**27.2951**	**51.8237**
Multi	**41.2555**	**< 0.001**	**28.4610**	**54.0501**
g-comp	**41.2555**	**< 0.001**	**26.0594**	**55.3315**
PSM	**42.6733**	**< 0.001**	**29.8507**	**55.4960**

Postoperative medical complication	OR	*p* val	LL	UL
Raw	0.8640	0.6289	0.4620	1.5235
Multi	0.8460	0.6325	0.4124	1.6369
g-comp	0.8520	0.6204	0.4519	1.6061
PSM	0.8599	0.6403	0.4418	1.5800

Postoperative surgical complication	OR	*p* val	LL	UL
Raw	0.8125	0.4357	0.4708	1.3441
Multi	0.8307	0.5492	0.4409	1.4935
g-comp	0.8327	0.5362	0.4663	1.4873
PSM	0.7379	0.3018	0.4036	1.2864

Postoperative day of first bowel movement	Beta	*p* val	LL	UL
Raw	**− 1.0770**	**< 0.001**	**− 1.3432**	**− 0.8108**
Multi	**− 1.1102**	**< 0.001**	**− 1.4116**	**− 0.8088**
g-comp	**− 1.1102**	**< 0.001**	**− 1.3426**	**− 0.8733**
PSM	**− 1.1762**	**< 0.001**	**− 1.4692**	**− 0.8831**

Length of Hospital Stay (days)	Beta	*p* val	LL	UL
Raw	− 4.7399	0.1441	− 11.1036	1.6238
Multi	− 4.6812	0.2195	− 12.1613	2.7990
g-comp	**− 4.6812**	**0.0064**	**− 9.1443**	**− 1.6886**
PSM	− 4.7984	0.1462	− 11.2754	1.6785

Reoperation	OR	*p* val	LL	UL
Raw	0.8965	0.8134	0.3287	2.0830
Multi	0.6198	0.4252	0.1655	1.8263
g-comp	0.6223	0.2942	0.2565	1.5096
PSM	0.6562	0.4548	0.1864	1.8007

Complication after DLI closure	OR	*p* val	LL	UL
Raw	1.8717	0.0765	0.9019	3.6540
Multi	1.4855	0.3341	0.6378	3.2246
g-comp	1.4753	0.2919	0.7159	3.0405
PSM	1.1685	0.6896	0.5208	2.4371

Bold indicates statistically significant results (*p* < 0.05)

*Raw* Unadjusted comparison, *Multi* Multivariable models, *PSM* Propensity score matching, G-computation, *OR* odds ratio, *p val* *p* value, *LL* lower level, *UL* upper level

For key secondary outcomes, minimally invasive approaches demonstrated faster recovery. Both robot-assisted (*β* − 1.18, 95% CI [− 1.65, − 0.64], *p *< 0.001) and laparoscopic (*β* − 1.11, 95% CI [− 1.34, − 0.87], *p *< 0.001) procedures resulted in a significantly faster return of first bowel movement compared to open surgery. While most adjusted models showed no difference in length of hospital stay, G-computation analysis revealed a significantly shorter stay for both robot-assisted (*β* − 4.13 days, 95% CI [− 10.01, − 0.62], *p *= 0.043) and laparoscopic (*β* − 4.68 days, 95% CI [− 9.14, − 1.69], *p *= 0.006) cohorts compared to open. No difference was found in complications after diverting loop ileostomy closure. However, the overall diverting loop ileostomy closure rate was lower in the robot-assisted cohort. This discrepancy is an artifact of chronological bias rather than a clinical outcome. The open and laparoscopic cohorts encompass patients treated between 2005 and 2025, ensuring ample time for stoma closure. Conversely, the robot-assisted cohort comprises our most recent cases, performed exclusively with the Hugo RAS platform up to September 2025. Consequently, a large portion of this group has not yet reached our standard clinical interval for DLI closure.

## Discussion

This study presents a matched comparative analysis of open, laparoscopic, and robot-assisted ileal pouch-anal anastomosis performed for ulcerative colitis and indeterminate colitis in the biologic era. The results demonstrate that all three techniques are safe and effective when performed in specialized high-volume centers, with comparable short-term postoperative outcomes and low complication rates. These findings reinforce that surgical expertise and patient selection remain more critical determinants of success than the surgical approach itself.

Minimally invasive IPAA, both laparoscopic and robot assisted, has consistently demonstrated reductions in surgical trauma and recovery time compared with open surgery, findings reproduced in the present cohort [18–20]. These benefits, however, should be interpreted considering operative complexity, surgeon experience, and institutional resources. Laparoscopic IPAA, whose safety and feasibility have been well established [21], has traditionally represented the benchmark minimally invasive approach. Because pouch construction and double-stapled anastomoses were performed identically extracorporeally across cohorts, our comparative findings primarily reflect differences in the execution of the deep pelvic rectal dissection rather than variations in total IPAA reconstruction. The robot-assisted technique, in turn, represents a natural technological progression, developed to overcome the ergonomic and visualization limitations of laparoscopy, particularly during deep pelvic dissection [22]. In this series, the use of a novel modular robotic platform for IPAA resulted in perioperative outcomes equivalent to those of laparoscopic and open surgery, demonstrating that this next-generation system can be reliably adopted into reconstructive colorectal practice without compromising safety.

Operative time was significantly shorter for open procedures, consistent with prior reports [23]. However, the duration of robot-assisted IPAA was comparable to that of laparoscopy, indicating that the introduction of robotic technology did not prolong operative workflow even for naive robotic surgeons. The similarity between robot-assisted and laparoscopic operative times suggests that modern modular robotic platforms can achieve procedural efficiency early in the adoption curve when implemented in centers with established minimally invasive expertise. These findings contrast with earlier reports describing longer robotic cases and likely reflect improvements in operating room logistics, team coordination, and system setup. While robotic systems still require higher initial preparation time, this difference diminishes with standardization and familiarity, supporting their routine use without compromising operating room efficiency [24, 25].

Although the unadjusted 30-day surgical complication rate was higher in the robot-assisted group (23.9%) compared to the laparoscopic group (14.2%), this difference was largely driven by minor (Clavien–Dindo Grade I–II) complications, such as ileus. Furthermore, our robotic cohort incorporates our initial experience with the Hugo™ RAS system, inherently capturing the platform’s early learning curve (docking setups, robotic arm positioning, and surgeon adaptation). Although this early phase contributed to slightly longer operative times and a higher proportion of Grade I–II minor postoperative ileus, the platform demonstrated safety profiles comparable to mature laparoscopic and open approaches once adjusted for baseline clinical characteristics via PSM and G-computation. During this early adoption phase, patients were selectively chosen to mitigate surgical risk, resulting in a preference for younger patients, lower BMI, and non-hostile abdomens. We explicitly accounted for this selection bias by utilizing propensity score matching (PSM) and G-computation models to adjust for these baseline differences. Ultimately, the small sample size of our robot-assisted cohort is underpowered to detect statistically significant differences in these complication rates. Anastomotic leak rates remained low across all cohorts (2.2% robotic, 1.4% laparoscopic, and 1.4% open). Clinically, anastomotic leaks in completion proctectomy and IPAA are multifactorial, stemming from chronic tissue fibrosis or residual rectal stump inflammation following subtotal colectomy, microvascular compromise related to long-term corticosteroid or biologic exposure, and anatomic tension within narrow android pelvis. Because all patients received a DLI during completion proctectomy, no leaks resulted in systemic peritonitis, and all were managed successfully via conservative measures, CT-guided drain placement, or transanal endosponge therapy.

Open surgery continues to represent a cornerstone of restorative proctocolectomy and remains indispensable in specific situations. It offers proven long-term results, excellent pouch function, and durable quality of life outcomes, as confirmed by large multicenter studies. The open approach allows direct tactile feedback and broader exposure, providing distinct advantages in complex cases, such as reoperative pelvis, severe inflammation, distorted anatomy, or prior biologic exposure, where minimally invasive dissection may be limited [26–28]. In such circumstances, open surgery ensures procedural safety and reliable reconstruction, preserving fundamental principles of pouch creation and anastomotic integrity. Therefore, open IPAA continues to be a valid and resilient option that should not be regarded as obsolete, as proved by the similar morbidity to minimally invasive techniques after matching.

The choice of surgical approach should thus be individualized, balancing disease extent, pelvic conditions, body habitus, prior therapy and surgery, and surgeon experience [29, 30]. Robotic systems, such as the modular Hugo RAS platform adopted in this series, offer improved maneuverability and 3D vision, potentially enhancing precision in confined spaces. However, these advantages are counterbalanced by increased setup demands, particularly during the learning phase. Laparoscopic IPAA remains a well-established modality with reproducible outcomes and technical reliability, while open surgery guarantees consistent results in challenging cases. Accordingly, the three approaches should be regarded as complementary components of a modern surgical armamentarium rather than competing modalities [31].

The present study’s strength lies in its balanced evaluation of techniques during a period of expanding laparoscopic and robotic utilization. All three modalities resulted in comparable complication rates, highlighting that quality outcomes rely primarily on multidisciplinary care, surgeon expertise, and postoperative management. Minimally invasive techniques demonstrated the expected benefits of faster bowel recovery and shorter hospital stay, while open surgery maintained excellent safety and durability. The equivalence among these approaches supports the ongoing paradigm of tailoring surgical strategy to individual patient and institutional factors rather than a uniform technique preference.

Several limitations deserve acknowledgment. First, the retrospective design precludes causal inference despite robust matched analyses. Second, the relatively small robotic cohort (*n* = 46) reflects early platform adoption, leaving the study underpowered to detect subtle differences in low-frequency adverse events; larger multicenter studies are required to establish statistical non-inferiority. Third, the 30-day follow-up window, an inherent boundary of our database, restricts our conclusions to immediate postoperative safety and early recovery metrics. Long-term outcomes, including late pouchitis, pouch failure, anastomotic strictures, and functional or sexual outcomes, require evaluation in extended prospective registries. Fourth, the 20-year recruitment period (2005–2025) introduces temporal variability in medical regimens (e.g., introduction of novel biologics and small molecules) and perioperative care; although PSM, multivariable regression, and G-computation adjusted for biologic and steroid exposure, unmeasured chronological confounding may remain.

## Conclusion

In conclusion, open, laparoscopic, and robot-assisted approaches each demonstrate distinct clinical roles in completion proctectomy with IPAA and DLI. Minimally invasive methods provide faster bowel recovery and shorter hospital stays with preliminary safety and feasibility comparable to open surgery. While robotic surgery achieves procedural efficiency matching laparoscopy, larger underpowered endpoints require validation in expanded cohorts. Ultimately, selecting the surgical approach should depend on individual patient habitus, disease complexity, and institutional expertise.

## Data Availability

The data underlying this article will be shared on reasonable request to the corresponding author.
